# Variable Populations of Grapevine Virus T Are Present in Vineyards of Hungary

**DOI:** 10.3390/v13061119

**Published:** 2021-06-10

**Authors:** Emese Demian, Aliz Holczbauer, Zsuzsanna Nagyne Galbacs, Nikoletta Jaksa-Czotter, Mihaly Turcsan, Robert Olah, Eva Varallyay

**Affiliations:** 1 Institute of Plant Protection, Hungarian University of Agriculture and Life Sciences, Ménesi Road 44, H-1118 Budapest, Hungary; emese.demian@gmail.com (E.D.); holczbaueraliz@gmail.com (A.H.); Nagyne.Galbacs.Zsuzsanna@uni-mate.hu (Z.N.G.); Jaksa-Czotter.Nikoletta@uni-mate.hu (N.J.-C.); 2 Institute for Viticulture and Oenology, Hungarian University of Agriculture and Life Sciences, Villányi Str. 29-43, H-1118 Budapest, Hungary; turcsan.mihaly@uni-mate.hu (M.T.); olah.robert@uni-mate.hu (R.O.)

**Keywords:** grapevine virus T, foveavirus, grapevine, high-throughput sequencing, diversity

## Abstract

Grapevine virus T (GVT) is a recently described foveavirus, which was identified from a transcriptome of a Teroldego grapevine cultivar in 2017. Recently, we surveyed vineyards and rootstock plantations in Hungary using small RNA (sRNA) high-throughput sequencing (HTS), at a time when GVT had not yet been described. A re-analysis of our sRNA HTS datasets and a survey of grapevines by RT-PCR revealed the presence of GVT in most of the vineyards tested, while at rootstock fields its presence was very rare. The presence and high variability of the virus in the country was confirmed by sequence analysis of strains originating from different vineyards. In this study, we demonstrate the presence of GVT in Hungary and show its high diversity, suggesting that GVT presence may not seriously affect grapevine health and that it could have been present in European vineyards for a long time as a latent infection.

## 1. Introduction

Grapevines serve as the basis of the wine industry, and the table grape is an agronomically important plant. Many chemicals are used to protect these vines from pathogens, but without an efficient plant protection method against viruses they will still be exposed to them. Grapevines are able to be infected by more than 80 viruses or viroids [1].

While traditional serological tests and RT-PCR can only detect the presence of the investigated virus, new high-throughput sequencing-based metagenomics methods can disclose the presence of all pathogens in a sample. High-throughput sequencing (HTS)-based virus diagnostics of grapevines is widespread and could lead to the characterization of the viromes of vineyards [2] and vines showing various symptoms [3]. Moreover, the evolution in the bioinformatic pipeline makes it possible to assemble the genomes of previously unknown viruses and viroids using these data [4,5]. This evolution in sequence data analysis has led to the description of an increasing number of grapevine-infecting viruses.

Grapevine virus T (GVT) is one of the viruses discovered by HTS in recent years. The original description of this new foveavirus dates back to 2017, when re-analysis of published grapevine transcriptome data revealed its presence in an Italian Teroldego cultivar [6]. The available sequence of the putative virus induced new analyses and revealed its widespread presence in Slovakia and the Czech Republic [7], Croatia [8], France [9], and Germany [10]. These descriptions were mainly based on the re-investigation of previously deposited RNA sequencing datasets and validation by independent methods. These efforts led to the identification of 26 almost full length genomic GVT sequences. However, in most of these cases, the 5′ and 3′ UTR of the sequences are missing or are incomplete. Recently, the reference genome of GVT (NC_035203) and the originally announced GVT full-length genome (MF095096) were removed from the GenBank because they solely relied on HTS data. The only report of GVT found when the causative agent of a grapevine disease was investigated was published in China. However, in this report, the symptomatic plant was infected with other viruses, the presence of which could be connected to the symptoms, including: grapevine leafroll-associated virus 1, grapevine red globe virus, and grapevine Pinot gris virus [11]. GVT was also reported overseas in California, where it was identified in an introduced Italian Lambrusca and, later, from a Russian, Georgian, and Croatian cultivar with Italian origin, which showed no symptoms [12].

Recently, we surveyed Hungarian vineyards and rootstock fields for the presence of viruses using small RNA (sRNA) HTS [13,14]. When we undertook our bioinformatics analysis, the genome of GVT was not available. However, its widespread, latent presence in all investigated European samples prompted us to reinvestigate our sRNA HTS datasets for its presence.

## 2. Materials and Methods

### 2.1. Source of Data and Samples

Our deposited datasets in the NCBI GenBank GEO, GSE106240 and GSE130994, contain sRNA sequence data from 18 samples representing 15 vineyards, and samples representing 17 certified rootstock fields and two rootstock collections, respectively (Table S1). We used these datasets for the bioinformatics analysis. For RT-PCR analysis, we used the originally extracted RNAs and RNA pools of these surveys.

### 2.2. Bioinformatics Analysis

From the sequenced sRNA reads we built up longer contigs using a de novo assembler of CLC Genomic Workbench (CLCbio, Aarhus, Denmark) software (with default options: word size 20, bubble size 50, and simple contig sequences and min 35 nt length), and searched for GVT-specific contigs. For BLASTn analysis, we used the sequence of the retracted reference genome (Supplementary Material S1).

Small RNA reads (both without and with redundancy) were directly mapped to the GVT reference genome. In the latter case, we calculated a normalized RPMR (number of redundant virus specific reads/million sequenced reads). Consensus sequences of the sRNA reads were prepared (extract consensus function of CLC) and, with the classical sequence analysis function of CLC, the coverage of the genome by virus-specific sRNAs was calculated as a percentage (Tables S2 and S3).

### 2.3. RT-PCR Validation

The cDNAs were synthetized using random primer and the RevertAid First Strand cDNA kit (Thermo Fisher Scientific, Waltham, MA, USA) from the RNA of individuals or the pooled RNAs representing the vineyards (the same RNA which was used to prepare the sRNA sequencing libraries). The PCR amplification was done by Q5 DNA polymerase (New England Biolabs), using two different sets of primers (Table S4). Primers designed by Glasa et al. [7] to amplify the 903 nt long parts of the viral genome were redesigned according to the sRNA sequences present in our samples, making them able to amplify a slightly longer (904 bp) product. A parallel PCR, using degenerated primers from Diaz-Lara et al. [12], which were optimized to amplify diverse variants of the virus, was also conducted.

### 2.4. Phylogenetic Analysis

To investigate the phylogenetic correlations of the GVT variants present in the country, we cloned the amplified 904 bp long product to a pJET vector (Thermo Fisher Scientific, following the manufacturer’s recommendations). One clone from each positive vineyard was Sanger sequenced and their sequences were deposited into GenBank (see Table 1 for their accession numbers, and their nucleotide and amino acid identity according to the GVT reference genome). Rootstocks had different geographical origins, which is why we did not include the corresponding isolates in the phylogenetic analysis. Phylogenetic analysis was conducted using MEGA7 and the neighbor-joining method.

## 3. Results and Discussion

To determine whether GVT is present in Hungary, we re-analyzed our sRNA HTS data generated in surveys of vineyards and rootstock plantations [13,14]. To our surprise, we found GVT-specific contigs in only three samples from the vineyards: 9_SZHT (Furmint vineyard), 13_BV (five ancient varieties in a collection), and 18_MK7 (Teleki Kober 5C rootstock cultivar sampled in the vineyards) (Table S2), and in none of the rootstock libraries (Table S3). The GVT genome shows high variability, so it is possible to lose hits when a strain showing low homology to the reference genome is present in the sample. To overcome this problem, we prepared a consensus GVT sequence using 26 full-length GVT sequences available in the GenBank (the same ones which were used for primer sequence optimization [12]) and blasted the contigs of the libraries against it. Two additional hits, 12_DF and 14_AD, one from a Furmint vineyard and the other from a rootstock plantation, were found. Direct BLAST of these contigs showed that the one presented in 14_AD showed 100% similarity to a *Vitis vinifera* sequence, which is a false positive. It is possible that during the GVT genome assembly, a read of the host was mistakenly incorporated into the virus genome. During our previous work, we noted that the presence of the contigs did not always coincide with the presence of a virus, which is why, using both the GVT reference genome and its consensus sequence, we directly mapped the sRNA reads of the libraries (map to the reference function of CLC), both with and without redundancy.

During our previous surveys, we found that the presence of the virus could be validated later if, besides the presence of a virus-specific contig, the RPMR was higher than 200 and the coverage of the genome was higher than 60% [13,14]. In the case of GVT, the number of virus-specific reads was very low and never coincided with an acceptable coverage. These results indicated that, in contrast to the reports from Slovakia and the Czech Republic [7], GVT is not abundant in Hungary.

During our previous surveys, we found that detection of another foveavirus, grapevine rupestris stem pitting-associated virus (GRSPaV), the closest relative of GVT, failed using sRNA HTS in most cases. The number of GRSPaV-specific sRNAs was very low in the infected plants [13,14], while virus-specific RT-PCR could detect its presence. To test if GVT was present in additional samples, in spite of the low level of GVT-specific sRNAs, RT-PCR with GVT-specific primers was conducted, amplifying part of its coat protein.

**Figure 1:**
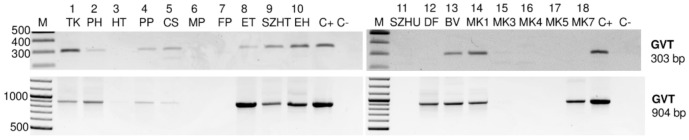
Validation of sRNA HTS by RT-PCR of the vineyard pools for the presence of GVT. Amplification was done using two different sets of primers, amplifying the 303 or 904 bp region of the coat protein coding region. Sample numbers, together with the vineyard codes, were used. M-GeneRuler 100 bp Plus, C-/C+ negative, and positive controls using water or cDNA prepared from a virus infected plant.

The results of the amplification using the RNA pools of the vineyards revealed the presence of GVT in more samples than the sRNA HTS did (Figure 1) (Figure S1).

In addition to 9_SZHT, 13_BV, and 18_MK7, we detected a virus-specific product in 1_TK, 2_PH, 4_PP, 5_CS, 8_ET, 10_EH, 12_DF, 14_MK1, and 15_TC. Results with different primers correlated quite well, but in 12_DF and 18_MK7 we obtained the product only when amplifying a longer product (Figure 1).

To reveal how widespread the infection is at different vineyards, sampled vines were individually tested for the presence of GVT (Figures S2 and S3, Tables S4–S6). This test showed a widespread presence of the virus at the vineyards, revealing that more than 93% of the tested plants (59/63) were GVT infected (Figure S2 and Table S5). In the rootstock fields, only two infections were found and GVT presence was also rare at the rootstock variety collections: 1/34 and 10/33 at 11_P and 15_TC, respectively (Figure S3 and Tables S5 and S6).

**Figure 2:**
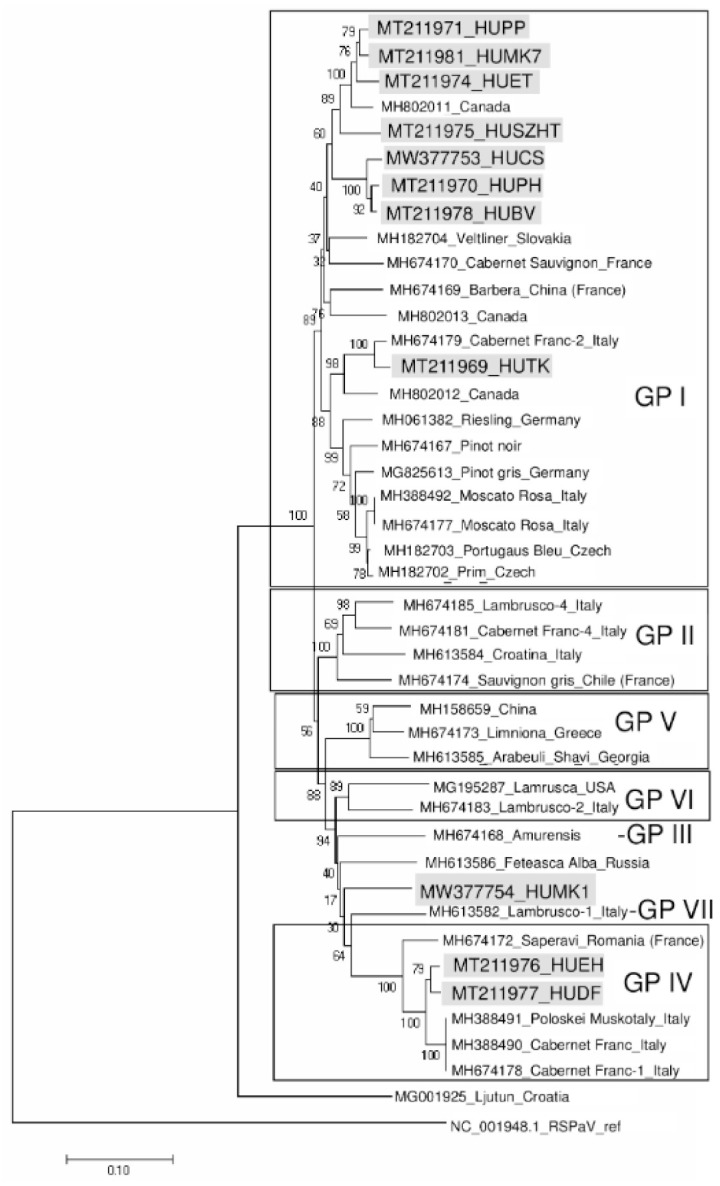
Phylogenetic relationship of GVT isolates. The evolutionary history was inferred based on the nucleotide sequence of a 904 bp RT-PCR product (coding for the coat protein) using the neighbor-joining method. The percentage of replicate trees, in which the associated taxa clustered together in the bootstrap test (500 replicates), are shown next to the branches. The tree is drawn to scale, with branch lengths in the same units as those of the evolutionary distances used to infer the phylogenetic tree. The analysis involved 43 nucleotide sequences. Their GenBank accession numbers are indicated. Hungarian isolates are marked with grey boxes. GRSPaV reference genome was used as an outgroup.

The low coverage of the GVT genome did not allow us to use the sRNA datasets to see how different the isolates were, and is why cloned, amplified RT-PCR products from each field were sequenced. The variants present in Hungary showed high variability, 82–89% nucleotide and 92–96% amino acid identity with the Italian Teldorego isolate, suggesting their different origins (Table 1). Previous analyses of GVT full-length genomes and CP coding regions have demonstrated the existence of seven clusters of isolates [9]. Phylogenetic analysis of the Hungarian variants showed that they cluster in Group I or the most distantly related Group IV, while the HUMK1 variant did not group with any of the previously identified groups (Figure 2).

DF, EH, and MK1 are vineyards in Tokaj, in the northeast part of the country, planted with Furmint, an ancient Hungarian cultivar [13]. The DF and EH plantations are more than 100 years old, while we have no information about MK1′s age. In Group IV, MH388491 represents a Hungarian breed: Poloskei muskotaly (bred in 1967), suggesting this clade could represent GVTs that have been in the country for a long time. Other Group I GVT variants were collected from different widely grown cultivars (including one additional Furmint, SZHT), which could originate from the international market and were planted much later. Vineyards where GVT was not present in the sampled plants were planted recently (within the last 10 years).

## 4. Discussion

Re-analysis of RNA samples from our previous surveys revealed high prevalence of GVT in the country. Based on studies on GRSPaV, foveaviruses have no identified vectors [15] and could be maintained during vegetative propagation. If this is true, GVT was present in Europe for a long time and could have been disseminated by vegetative propagation of the host. Elimination of foveaviruses from grapevines, as was shown for GRSPaV, is very challenging, and the methods used to do so show different efficiencies [16,17]. Meristem culture-based elimination could not 100% eliminate GRSPaV or GVT, which could explain their almost even distribution in the infected cultivars. However, chemotherapy and somatic embryogenesis-based methods gave better results for GRSPaV and for both viruses, which could be optimized in the future if new regulations target foveaviruses [16,18].

GVT was only described recently, and it can be present in grapevines without any visible symptoms. Its European origin is highly probable, as it has been identified only in European cultivars. The vineyards of Europe were almost totally replanted after phyloxera epidemics. However, *Vitis sylvestris* species in the wild flora could survive. Their virome can reveal information about the presence of endemic viruses. Investigation of the virome of a *Vitis sylvestris* population in Switzerland revealed the presence of a foveavirus, grapevine foveavirus A (GFVA–MN553040) [19]. GFVA is a highly divergent GVT isolate, or could even be a distinct foveavirus. Comparison of the CP coding region of Hungarian GVT isolates to the CP of GFVA showed higher than 80% amino acid identity and higher than 90% positivity, supporting the close relationship of GVT and GFVA (Table 1). This finding suggests that the origin of GVT may be from the endemic natural flora, with a common ancestor with GFVA, but this question needs further investigation in the future.

## 5. Conclusions

High-throughput sequencing techniques offer the possibility of gaining sequence information about previously unknown viruses. A recent paper, investigating the biological information in publications describing new viruses of fruit trees, revealed a big gap in their biological data, including host range studies and symptom descriptions, which could affect their correct risk assessment [20]. This is the knowledge that is still missing and should be detailed for GVT in the future. Currently, we can only show that GVT is widespread in vineyards, but it is not possible to directly correlate any deteriorating symptoms to its presence.

Latency and the symptomless nature of GVT infection, together with high variability of the genome, result in uncertainty in the viral diagnosis. The question of regulating GVT is open, but more information is needed about how GVT affects its grapevine host to make a sensible decision.

## Figures and Tables

**Table 1:** GVT sequences, which were deposited into NCBI GenBank, together with their accession number. Nucleotide sequences of the partial sequences were pairwise compared by BLASTn algorithm to both the GVT reference genome (Supplementary Material S1) and the GFVA sequence (MN553040). This pairwise comparison was done by BLASTp for the CP coding part of the partial sequences, and for calculating the identity and positivity in the amino acid percentage.

Library	Isolate	GenBank Identifier	Identity to GVT at Nucleotide Level %	Identity to the GFVA at Nucleotide Level %	Identity/Positivity to the CP of the GVT at Amino Acid Level %	Identity/Positivity to the GFVA at Amino Acid Level %
TK	HUTK	MT211969	86	78	94/96	85/93
PH	HUPH	MT211970	89	77	96/98	86/93
PP	HUPP	MT211971	88	78	96/98	81/89
CS	HUCS	MW377753	88	78	96/98	82/89
ET	HUET	MT211974	87	78	95/98	85/92
SZHT	HUSZHT	MT211975	87	78	95/98	83/91
EH	HUEH	MT211976	82	77	93/96	82/91
DF	HUDF	MT211977	82	77	92/95	82/91
BV	HUBV	MT211978	89	76	96/98	85/93
MK1	HUMK1	MW377754	84	77	94/97	85/91
MK7	HUMK7	MT211981	88	78	96/99	81/90

## Data Availability

The sRNA HTS datasets analyzed can be found at NCBI GEO: GSE106240 and GSE130994.

## Supplementary Materials

The following are available online at https://www.mdpi.com/article/10.3390/v13061119/s1: Tables S1 and S7 spreadsheets, Supplementary Figures S1–S3, and Supplementary Material S1, containing the retracted sequence of the originally described GVT genome (MF095096), which was temporarily the reference genome of GVT (NC_035203).
