# Supplementary material for: Variable Populations of Grapevine Virus T Are Present in Vineyards of Hungary
Source: Viruses. 2021 Jun 10;13(6):1119. doi: 10.3390/v13061119 (PMC8230486; doi:10.3390/v13061119)
Supplement: Supplementary file 1 [file viruses-13-01119-s001.zip › Supplementary Material S1 to Demian el al.pdf]

File S1 to

Communication

Variable populations of grapevine virus T are present in vineyards of Hungary

Emese Demian[1x], Aliz Holczbauer[1x], Zsuzsanna Nagyne Galbacs[1], Nikoletta Jaksa-Czotter[1], Mihaly Turcsan[2], Robert Olah[2], Eva Varallyay[1*]

*corresponding author: Varallyay.Eva@uni-mate.hu, Hungarian University of Agriculture and Life Sciences, Szent-Györgyi Albert Street 4, 2100, Gödöllő, Hungary

```
>NC_035203_GVT Grapevine virus T isolate Cho replicase (ORF1), TGB1 (ORF2), TGB2 (ORF3), TGB3 (ORF4), and CP (ORF5) genes, complete cds.
CGCGGATAAACAACAGAACCCGAACGGCCATACATCAAAGTGGTACAACTACTTTCACGTAATGGCTCTTCACCA
CCGTTCTGCTGCTGCTGAGGTGCTCGCCCGACTCACCTCAGATGAACAATCTAGAGTTAGTGCTCCAACAGTAAA
ATCCTTGTGTGATGATGAGGAACAGGCCGGTGGTTTTTTTAACTACAGCCTATCACAGAAGGCGCAGGAAAAATT
GGAGCTCTCTGGTATCTATTTGAGCCCACTTAGTTATAAGCCACATTCCCACCCTGTGTGCAAAACTCTAGAAAA
TCATATCCTCTACAATGTATTACCTAGTTACTTAGACAATAAGTTTTATTTTGTAGGGATTAAAAATTTCAAATT
AGATTTTATTAGAAGTAGAAATAAAAGGTTGTCAATGGTTGAATCTGTAAATAGGTATGTCACCTCATTAGATAG
ATCCAGGTATTGTAACGAGCTAATCCATAGGAGTTCAGCATCGGATCTACACCTTGCCAGAAAGAGAGGTTTCAA
TGGATTGCCAACCCTAGCAAACCTTATTCCCCCACTTGAGAAGAAAGGGGCGGAATTCATCTTTCTCCATGATGA
GTTGCACTACTGGTCTCCTTCCTGTGTTATTAAATTTTTGGAGGCCATTAAGCCAACAACTGTGCTAGCCTCTAT
TGTTGTCCCTCCAGAGTTGCTCGCCAAATCCAAAGAGAGTCTTAATCCATGGGCGTACAAATTTAAGATCATTAA
GGATCGCCTGGTCTTTGCCCCAGATGGAGTTTGGGCTGAGGCTTATGATCAACCCTTGCGATGCAAGTACTTGCT
CAAGACAAGGCGGTTTCTGCTTGGTGATGGCTCTGTGTATAGCGTGGATATTGTTCATTCTAAATTCTGCCACCA
TCTGATAAGCATTACACGCGGGGAATCATTGAATCAAGATCGCAGGTTCTTTGGAGATTTTGACGCAATCGGGCT
TGGTGCCTTGGGCAAGTTGAGTAGGAATGTCAAGCATTGCTATCCAATAAGTGTTCAAGTAGTCACCAAATTGTA
CTTGTACCTCATGACCTTAAAGAAGCCCGATGAGGAATCAGCTGAAGCCAAACTAAGGCAGCTAATTAATGATCC
AACTGGGAGAGAGATCAAATTCGCTGAGAGTTTCTCTAGACTGATTTTGAAATGCAAGAAAGAGAGCTCACTGTT
TCTTCCAAGCTTGACAAAAGACTGCAAAAATTTTTTAATAGGGCTGTTCCCAGGTTTTATATCAAGGATGTTCCA
GATCTACAAGGATAAGTGTCTGAGTGACTTCATTTATGATCTAGAACCATTTAATTTTTATGTTGACACCGTTGA
TGTTAAAGGGGACTTCTCTGATGAATTTGAGTTTTGGGACTTACATGATTTACTTGATTCATTTGATGTGAGTGA
AGGGATTGATGATATGAAAGTCACCATAAAGAATTGGTGGAACACAAGGTCACCACAGAGCTACTTCATCGGCGG
GAGAGAGTGGGAGAGTAGCAGTTCAAAATTTTACTCTAAGTTCCAGGCTTTCGAACGAGTTGGGGACCTTTACCG
CTACCTCTTACAAGATCTTAGTCATGATATGGTGAATGAAGAAGTTGTGGTTCGGAGATTGCGGGACTTTTTGAG
AAGAAGGGGCACTGAGAGTCTTGCATATATGATATCGTGCAATTTGGATATCACAGCATTGAAGAAACTGTCTT
TAACTGGACCTTGGAGGCCAGGAAGGAGAATTGGCTGCGTGTGGTGCGTCCGCAGTGGTTTCTACTTGTTGGGAG
AATTAACACTAAGTTCATTTGTGAATCGAGTAGTTCCATCTCCAACCTGTCTGTTGAAAGTGTCAATCTTTATCG
TTCAATTGTGAAAGAGGTTGAAAAGTTCAAATTAGAAAGTCTTGATAGAGTGTTTTGGAGGCAAACTAGTTCCGC
CATGGAATTTGGAGGGCCAAAGTTTGAGATAAGCGAAGAACCTGTAGTGGAGGTTGAAGAACAATGTGCCCCTGA
AGAGGTGATTGATGTTGGTTCTAAACAAACCTCAGCCGTCATTCATGCCGAAAGCCAAGCTACGGCTCCAAGTTA
CGTTGACTGCACCTCGGTTAGCATTGATGAAATCAAGATTGACATTGGAAATTCAAGTGAGCTGAAATTTCATTA
TGGAGTGCTGAAGTTTCCTGATGTCTTGAAGGGGCGCAAGGCCGGTTTTTATGCAAAATCCGGAATTAAATCCTA
TGCTTATAATGGTGGAAGTCATGACAGTATGGGATGGCCCAAAGCACTTGATGAAATCCTTAGGCTGGTTGATGG
CGGTCTATATTACAACAGTTGCTTGGCACAAAGGTATGATGGTGGAGCCTCAATTGGGATGCATTCTGACAATGA
GAGGTGCTATGCTGTTGGGCATAAGGTGCTCACTGTCAACATGCTTGGGTCATGCCAATTCTACATATGCAGGAA
GCAAGATCGCAATCTCAAGGAGCGGCACATAAATTTTAGGCTCAGAGAAGGTGATTATTTTGAGATGCCACGTAA
TTTTCAGGAGAATTATCTGCACGGAGTTCGTGAAACAAGTGATGGTCGAATATCTTTGACCTTCAGGAGGCAGCT
AGTCCCTGATTCGGGTGATGATGAGGAGGAATGCAGCCCATTTGCATTCGGCAGCATTGACAGGATTGCTGGCCG
AAAATTTTGGCCTGAATGCTCAAATTATTTGGCAGCGATGATTGATGGTAGTGGCAAGGGAAGAGAATTGGATCT
TTGTGATTGTTTCTCTTGTCCAAATCACTTTCGGCATGCCAAGAACATTGACGGGATAAGAAAGGTTTTTTCAGC
CACTGGGTTGGCAGCTGGAGACAGAGTTCTGGTTTTAAATTCAAAATCAAGTAAGGCAATTTTGGAAAAATTCGA
```

AAGGTTGAGGGTTACAATTGCTGTTTTCTCTGGAAAGATTGAGTTTCTGGATGGTCTTGTCCCCGGGATTGTGAG
TGATGGGGACCTCAGGGAGGTGAAGAACTTTTCAGTGCAGAATCTGAAAGTTGAGCTGGCAGTCATTATTTTCCG
TGGTTCAACAGTATCTGGCTCGAAGGTGCGGGTGCACAGAGGGGAATGCAAGTTCACTGGAGCGAGTGAGGTATT
ATTTGGCTGCAATTTGATTGCTACTGATGAATATTCAATTGGAAATTTTAACTTATTTCAAGTTCCAGCTGACGG
TAATTGCTTCTGGCACTCTGTGGGCCATTTGATGAATTGCAATGGCCTTGATTTGAAGAGAGGTGTTTTTAATAA
ATTTAGAAGCTCAGGTTTGGAATCAGTGAAGCTTGAACATCAGTTGGAGCCAAATGTTTTTGCAGAGAATGAGGT
AATTGCATACTTCTGTGGTATGCACAAAAGGCGACTAATAATATTAACCCCAGAGTATGATGTTGAGTGGAATTT
TGGTGAGAAGAACTGGCCATTACTTGGCTTTCTGTGTCTCGTGCAGCAGCATTTTCAGCCATGCGCGCCAACTAA
TGGATGCATGATTAGGGCCGTCGCTGAAGCCCTTGGAAAGCGAGAGGTTGATGTACTTAATTACCTGTGTCAGGA
TGCAACCAGACATATATTTGAAGAAGTTTGCCAGGGCGCTGGTCTCAACTTGATGTATTTGGAGGCCGTATTTGA
AGCTTTTGACATTTGTGCTAAATGTGACTTTGGGGACAGAGTTGAAACATTTAATAACTCAGGCTCAGTAGTTGC
ATTGTTCGATGTTTCAGCTGAACACATAAGGTGTGTGCCGAAGATTTCTGACAAGCCCATGAGTGTGAGGGTTGA
TGCCATGAGGAGTGTAAGAAAGTCTGAAATTGAGATGCTTGGCATGAACGGAACTTGGATTGAGTATGAGCCACA
GGTGGCTAGAGCTGAAAAATTGGAGAAGTGCTTGCTTGCTGGTCTTACTGGAGTGATGTGCAGTGAGAGGTATGA
CAACCAAAAAGCTTGGCTGGATAACATTGCCAAAGATCAAAGGGGTAAACGCAACATTATGGCCATAATGGGTAC
TTTTGGAAGTGGAAAGAGTCATGTTTATAAGACTTTCATGACCCATGCAGTTGGAAAATTTGTAACATTTGTGTC
TCCTAGAAGGCAGTTGGCTGATAGCATTAAGGATGATCTGGGTCTTTATAAGGATAAACTTGGGAAAACATTGAG
AAACAAATCCCAAGAGGGGTGGAGCGTGGTTACTTTTGAAGTTTTCTTAAAGACCATTCCACAACTTAAAAAAGG
GCATGTGGTCATTCTTGATGAAATTCAGCTATTTCCGCCTGGCTATCTGGATCTTTGCCTACTCGTGTCAGATAA
GTCCTGCATTTTCTCAGTTGCAGGCGACCCTTGCCAAAGTGGCTATGATTCAAAGAGCGATAGAAACATTTTGGG
GGCTCTAGATTCTGATATATTTGAGCTGTTGAGGAACAAAGAATACTCTTACAATGTTGAGAGTAGAAGGTTCAA
AAACCCTATGTTTGAAGGTAGATTACCATGCCGGTTTTTGAAGGGATCATTGCACGAAAAACATCAGGATTTTGT
GGTGTGGAAGTCTATGGAGGATTTGGTCTTTGGGGAGAAATTTAAGATCGATGCATTCCTGGTGAGTAGTTTTGA
GGAGAAAAGGATAGTATGGTCACATTTTGGATCTGCAGTCACCTGCCTCACATTTGGGGAATCAACAGGTCTGAA
TTTTAACGAGGGAGTTGTGTTAATAACTTATGATTCAATTCAGACAGATGACAACAGGTGGCTAACTGCATTGTC
GAGGTTCAGGATGAACATCCATTTTGTCAATTTATTGGGGATCCCCCTGGAAAGGGCAATAGGACATTTCGCTGG
CAAACCACTTTATCACTTCATGTCTAAGAAAAGTGGAGAAGCAATCATACTTGACATGCTACCTGGCTGCCCGAA
GTTCAAAAATGGATTCAGCATCTCTATTGGTAAGGATGAAGGAATTAAGGAGACAAAACTTGCAGGTGATCCATG
GTTAAAGACAATGTTATTTTTGGGTCAAAATGAGGATATTGAAATAGAAGAAATGGAGGAGGAATTTAAAAATGA
AGAGTGGTTCAAAACTCACTTACCTATTACCTCCATGGAATGCTCAAGGGCTAGGTGGGTCGGAAAACTTGCACT
GAGGGAAAGTAGAGAGGTGAGATGTGGAATGAATACCACTGAGCAATTTAAGGATGATCACCATGACCTTAAAGG
GGTTTTGATGACAAATGCATGCGAGAGATATGAATCTATATATCCAAGGCATAAAGGCAATGACAGCGTCACCTT
TTTGATGGCTGTTAAGAAGAGGTTGCGTTTTTCTAGTGCTGCAGTGGAGGAAGCATTGCTCAGAAAGGCTATGCC
ATTTGGGAAGTTCCTTCTTGGAAAATTCTTGAAAAATATTAAATTGAAAAGGAACCACAATCCACGAATGATGGA
GGAATCAGTGGCGGAATTTGAGGAGAAGAAGACCAGTAAGAGTGCTGCAACAATTGAGAATCACAGCGGGCGATC
ATGCAGGGATTGGTTGAATGACGTTGCCTTGGTTTTCATGAAGTCACAACACTGCACGAAGTTCGACAATCGGTT
TAGAGATGCCAAGGCAGGTCAAACTCTTGCCTGCTTCCAACACTCCGTGTTGTGCAGGTTCGCGCCATACATGAG
GTACATTGAGAAAAAGGTTCTTGAAGCCTTACCTGAGCGATTTTACATTCATTCTGGGAAGGGTTTGGATAGTCT
AAAGGCCTGGGTGGTTGCAAATGATTTTTCGGGGGTGTGCACTGAGTCCGACTATGAAGCCTTTGATTCATCTCA
GGACCATTACATTCTGAGTTTTGAAATTGAGCTGATGCGGTATTTCGGGCTTCCTAATGATCTAATCCTTGACTA
TCAGTTCATAAAGACTCATCTGGGCTCCAAGTTGGGGAATTTTGCAATAATGCGGTTTACAGGTGAAGCTTCAAC
TTTTCTGTTCAATACGCTAGCCAACATGCTATTTACCTTTTTAAGGTATGATTTAACTGGTGGCGAGAGCATCGC
ATTCGCTGGAGATGACATGTGCGCGAACAAGCGTCTGAGGGTGACTAAAGAGTATGAGGATTTCCTAACTAGGAT
TCGGCTGAAGGCTAAGGTGCAATACGTAGCTAGCCCGACCTTTTGTGGTTGGTGTCTATTCAAGAATGGGATTTT
TAAGCGGCCACAGTTAGTCATGGAGCGAATAAACATCGCCAGGGAGATGAACAATTTGGACAATTGCATAGACAA
TTATGCAATTGAGGTTTCATATGCCTATTCTTTGGGTGAGCTTGCAGTGGAGGTGATGAGTGATGAGGAGCTCGA
GGCTCATTATAATTGCATTCGCTTTTTGGTGAAGTACAAGAATAGGATGAAGAGTTCCATTAAGGAGCTCTTCTA
GTTAGTAGTGCTAGTCAGCAGTTAGGTGAAGCTTGTTCATTGATTTATCTTGTGTGATGAATAATTTGGTTTCTG
CTCTTGAGTTGTTTGGGTTTGTTAGAATTAGCGAGGAAGCTAGATATCCCTTAGTCATTCATAGTGTCCCTGGAA
GTGGAAAGACAAGTTTGATCAGGTCTTTGATTAAGTTGGACGGTGATTTTGAGGCATTTACAGCTGGAGTGCCTG
ACACACCAAACCTTGAGGGGTGCTACATCAGATCCCACTTTGAGGGTTGTGCTTCAAATAAAAAGTTGAGCATTC
TGGACGAGTACCTCACAGTTGAGAATTGGGAAGGGTTTGACGCACTGTTTTCTGACCCTTACCAGAACGATAAGA
GCCCTCTCGTGGCCTCCTACGTGTCTAAGAAGACCAAGAGATTTGGAAAGTCAACTTGTGCGTACTTAGCAGATT
ACGGCTTTGAAGTAGAGAGCGAAATTGAGGATTTAGTGGTCAGGGGTTCACCATTTGAGCTCAAAGTTGAAGGGC
AATTGATTTGCTTTGGGAAAGCTGCAGTCGAGCTTGCATTAAGTCACTGTGCAGAGTTCAAATTGCCTTGTGAAG
TAAGAGGTTCCACATTTGATGTGGTTACGCTGTTGAAATCAGAAGAGCCAAGCTCAGTGAACAGGCATCTGTTTT

ACGTCGGATGTACAAGGCACAGGAAGAAGCTTATAATTCTTGAGTAATTGATGTCATTTCAGCAACCAGCCAATT
GGTCTAAATCACTAAGGCCTGTGTTGATTGGTGCCGGAGTTGCAATTGTGATCCATTTTCTAAGAGAAAGTAATC
TGCCACATGTTGGAGATAACCTGCATCACCTCCCACACGGAGGAAGTTATCAGGACGGAACAAAGAGAATAACCT
ACTGTGGTCCTAAGAAAGAATATCCAGGTACTGGTCTCTTTCAACTTGGGAATTCCCAAACTGCAGTTGTGTTGG
TTCTTGGCTTGTCACTCTTGATCTATGCAGTGGAGAAGTTTGGCATTGGGACTTATAGGGGCTGTGCTTGCAACC
CTAATCCTTGCGTATGTAGGAGGTGAGAGAGGCGGCAGTTGCTTCATAAGGATCACCGGTGAGTCCGTCACTGTG
TCCAACTGTGGAAACCTTCAAGAGGTGCCAGAAGTGATCAAAGCGGTGAAAGGAGTGTGGTCCTCGTTAGGTGCA
GGGGTAAATTTTGATTTGGAAGGCAACTGCAGTTGAAATGGCGTCGAATGAAGAGCTGAGGAAGCTAGAGGAGAA
AAGGCAGAGGGACGAAGCCAAGACACTTGGGAAGGAAATAGAGGGTGCAGGAGGAAGCAGACCAGCATTGCCAGA
TTTGGGAACAGTAATTAAAAGGAAGAAAAAGCAGATTGAAAATGCTTTGAGCGCCAAAGTTAACGTGAAAGATCT
GATGGAGAGGGAATTTGGCCTCCAGTCAAACAATGTCATGAACAACAATGATATCCTTGCCATTTCTAGCATATT
TACAGAAGCAAAGATACCAGAAGATCTGCAGCAACATACTGCAATAGAGGTCGCTAGAATGGCTGTGGATGTTGG
CAGCTCAAAACATTCAGAATTCATGGGAAAGAGCGTTGTATGTGGGCATGACTTATCGGCAATTGTTGGCTACAT
AAAGGAAGTGACTACACTGCGCAGGTTTTGCATGTATTACTCCAAAGTGATATGGAACTTGCTTATCAAGGAAAA
GACTCCTCCCGCTAATTGGGCAAAGAAAGGGTTTAAAGATGAGACAAAATTTGCAGCCTTTGACTTCTTCGTCGG
TATCTTCGATGAAAGTGCATTGGAGCCGGAGGGGGGCTTGGTGAGGTCACCCACACAGAGTGAGCTTAAGGCAAA
TATTTCTGCTTTGGAAGTGAAGGTTTTCAGGCAAAAAATGAGTGAAGGTAACAGGTCTTTAAACTTGGGGGAAAT
AAGCGGGGGAGCCATGGGTGCTCCAGCTTACAATCCATTTGGGGGAGATTCATAAGTTTTAACTTTTGGTGTTTC
TGCTGATTAATGATCAGCACTTGCTGAAGCTTGAGAGTTGCTAGTTGGAACTCTTGCCGCAGTCAACTTTATAGT
ATGTGCGACACCCATTTCGATAGGGCAAATGTTCGCTGGCATACCGTACTAATAGTATGCTTCTATCTAACTGAC
GATCCTAAGACCTAAATAATTATATAAGGGAATCGTCAATAAATAAAAATGCGTTGAGAGTTGCTAGTTGGAACT
C
