# Supplementary material for: Variable Populations of Grapevine Virus T Are Present in Vineyards of Hungary
Source: Viruses. 2021 Jun 10;13(6):1119. doi: 10.3390/v13061119 (PMC8230486; doi:10.3390/v13061119)
Supplement: Supplementary file 1 [file viruses-13-01119-s001.zip › Supplementary_Figures S1_S3_to Demian et al.pdf]

viruses MDPI

Figures S1-S3 to

Communication

Variable populations of grapevine virus T are present in vineyards of Hungary

Emese Demian[1x], Aliz Holczbauer[1x], Zsuzsanna Nagyne Galbacs[1], Nikoletta Jaksa-Czotter[1], Mihaly Turcsan[2], Robert Olah[2], Eva Varallyay[1*]

*corresponding author: Varallyay.Eva@uni-mate.hu, Hungarian University of Agriculture and Life Sciences, Szent-Györgyi Albert Street 4, 2100, Gödöllő, Hungary

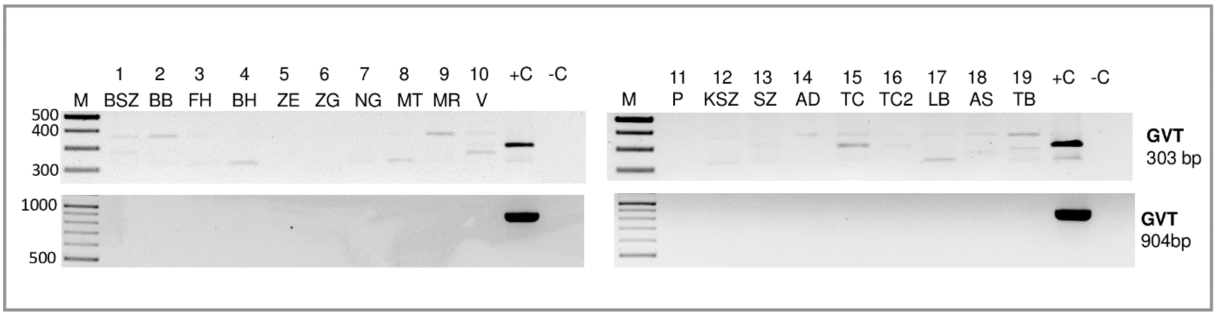

Figure. S1 Validation of small RNA HTS by RT-PCR of the rootstock plantation pools for the presence of GVT. Amplification was done using two different sets of primers, amplifying 303 or 904bp product. Sample numbers together with the plantation codes are used. M-GeneRuler 100bp Plus, -C/+C negative and positive controls using water or cDNA prepared from a virus infected plant.

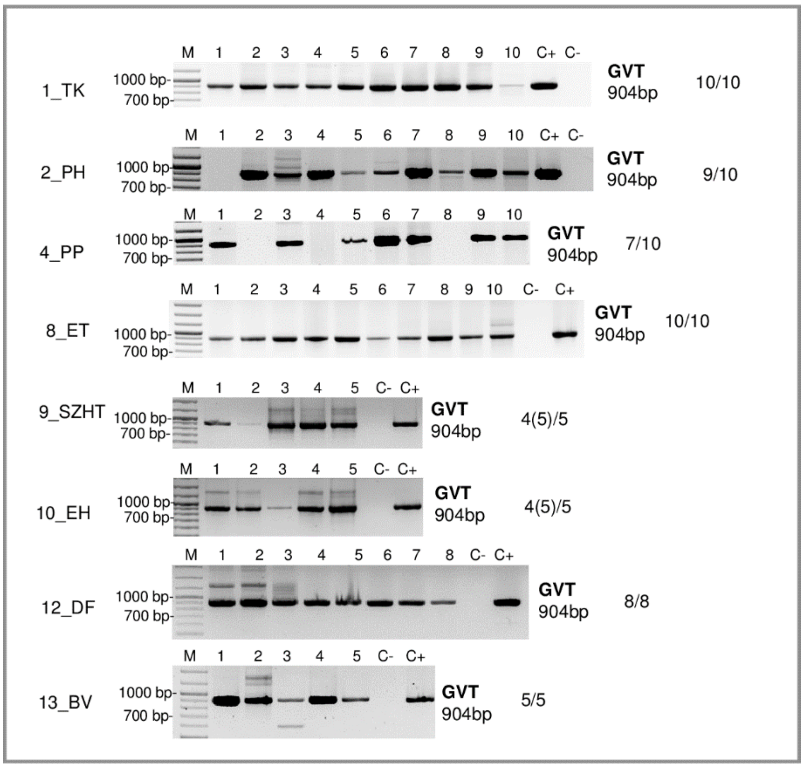

Figure S2. GVT specific RT-PCR test of individuals at the vineyards. Amplification was done using primers, amplifying a 904bp product. Sample numbers together with the plantation codes are used. M-GeneRuler 100bp, -C/+C negative and positive controls using water or cDNA prepared from a virus infected plant.

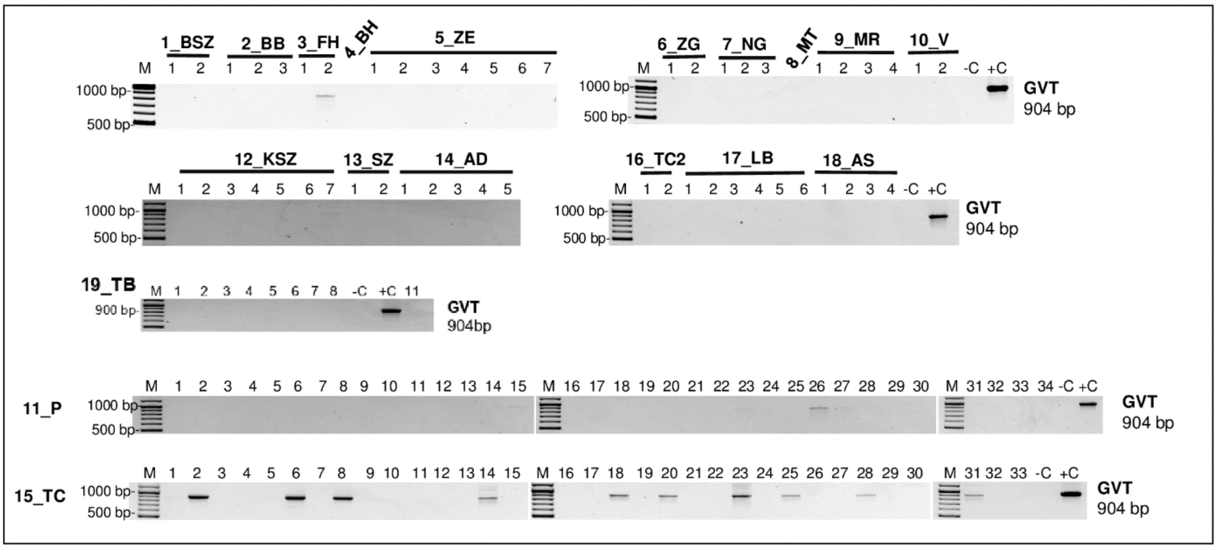

Figure S3. GVT specific RT-PCR test of individuals at the rootstock collections. Amplification was done using primers, amplifying a 904bp product. Sample numbers together with the plantation codes are used. M-GeneRuler 100bp, -C/+C negative and positive controls using water or cDNA prepared from a virus infected plant.
